# Evolution and Classification of Myosins, a Paneukaryotic Whole-Genome Approach

**DOI:** 10.1093/gbe/evu013

**Published:** 2014-01-18

**Authors:** Arnau Sebé-Pedrós, Xavier Grau-Bové, Thomas A. Richards, Iñaki Ruiz-Trillo

**Affiliations:** ^1^Institut de Biologia Evolutiva (CSIC-Universitat Pompeu Fabra), Passeig Marítim de la Barceloneta, Barcelona, Catalonia, Spain; ^2^Departament de Genètica, Universitat de Barcelona, Catalonia, Spain; ^3^Life Sciences, The Natural History Museum, London, United Kingdom; ^4^Biosciences, University of Exeter, United Kingdom; ^5^Institució Catalana de Recerca i Estudis Avançats (ICREA), Passeig Lluís Companys, Barcelona, Catalonia, Spain

**Keywords:** origin of eukaryotes, LECA, Holozoa, eukaryote evolution, chitin synthase, Smad

## Abstract

Myosins are key components of the eukaryotic cytoskeleton, providing motility for a broad diversity of cargoes. Therefore, understanding the origin and evolutionary history of myosin classes is crucial to address the evolution of eukaryote cell biology. Here, we revise the classification of myosins using an updated taxon sampling that includes newly or recently sequenced genomes and transcriptomes from key taxa. We performed a survey of eukaryotic genomes and phylogenetic analyses of the myosin gene family, reconstructing the myosin toolkit at different key nodes in the eukaryotic tree of life. We also identified the phylogenetic distribution of myosin diversity in terms of number of genes, associated protein domains and number of classes in each taxa. Our analyses show that new classes (i.e., paralogs) and domain architectures were continuously generated throughout eukaryote evolution, with a significant expansion of myosin abundance and domain architectural diversity at the stem of Holozoa, predating the origin of animal multicellularity. Indeed, single-celled holozoans have the most complex myosin complement among eukaryotes, with paralogs of most myosins previously considered animal specific. We recover a dynamic evolutionary history, with several lineage-specific expansions (e.g., the myosin III-like gene family diversification in choanoflagellates), convergence in protein domain architectures (e.g., fungal and animal chitin synthase myosins), and important secondary losses. Overall, our evolutionary scheme demonstrates that the ancestral eukaryote likely had a complex myosin repertoire that included six genes with different protein domain architectures. Finally, we provide an integrative and robust classification, useful for future genomic and functional studies on this crucial eukaryotic gene family.

## Introduction

The evolution of molecular motors was key to the origin and diversification of the eukaryotic cell. There are three major superfamilies of motor proteins: kinesins, dyneins, and myosins. The first two act as motors on microtubule filaments, while myosins function on actin (Vale 2003). Myosins participate in a variety of cellular processes, including cytokinesis, organellar transport, cell polarization, transcriptional regulation, intracellular transport, and signal transduction (Hofmann et al. 2009; Bloemink and Geeves 2011; Hartman et al. 2011). They bind to filamentous actin and produce physical forces by hydrolyzing ATP and converting chemical energy into mechanical force (Hartman and Spudich 2012). Both activities reside in the myosin head domain (PF00063). This head domain is accompanied by a broad diversity of N-terminal and/or C-terminal domains that bind to different molecular cargos, providing the functional specificity of the protein. Some myosins, such as myosins V and II, act as dimers that contact through their C-terminal coiled-coils, while others, such as myosins I, III, VI, VII, IX, X, XV, and XIX, act as monomers (Peckham 2011).

The identification of gene orthologs can be best accomplished by phylogenetic analyses, especially when complex architectures that are likely to undergo rearrangements are involved (Koonin 2005; Sjölander et al. 2011; Leonard and Richards 2012; Gabaldón and Koonin 2013). Thus, myosin phylogenetic analysis is important to classify myosin paralog families and identify the ancestry of different gene architectures. Previous efforts have been made to classify the myosin family and to reconstruct its evolutionary diversification (Richards and Cavalier-Smith 2005; Foth et al. 2006; Odronitz and Kollmar 2007), although information from some key eukaryotic groups that have recently become available were missing from all of these studies. Therefore, there is a need to revise schemes of myosin evolution using improved taxon sampling and phylogenetic methods. This is important both to update the classification of myosins diversity and also understand the origin and evolutionary history of the wider gene family. Moreover, a precise reconstruction of the ancestral eukaryotic myosin toolkit (along with that of the other motor proteins [Wickstead and Gull 2007; Wickstead et al. 2010]) has important implications for understanding the phylogenetic patterns and functional attributes of early eukaryotes (Richards and Cavalier-Smith 2005).

Previous analyses, using different genome datasets and different phylogenetic methods provided conflicting hypotheses on myosin classification and the reconstruction of this ancestral toolkit. For example, Richards and Cavalier-Smith (2005) provided a classification of myosins based on two criteria: phylogenetic reconstruction and analysis of protein domain architecture. They inferred that the last eukaryotic common ancestor (LECA) had 3 of the 37 defined eukaryotic myosin types, including Myo_head-MYTH4/FERM, Myo_head-SMC-DIL, and Myo_head-TH1. In contrast, Foth et al. (2006), in a study focused on apicomplexan myosins, defined 29 classes and did not infer an ancestral complement. Also based on phylogeny, Odronitz and Kollmar (2007) defined 35 different myosin classes, most with an extremely restricted phylogenetic distribution. To make things more complex, different authors have used different criteria for classification, leading to inconsistencies in the classification and nomenclature between studies.

In this article, we present a new evolutionary history and classification of eukaryotic myosins. We use a significantly expanded taxon sampling than previous studies, in which, for the first time, all major eukaryotic lineages are represented. In particular, we include data from four previously unsampled eukaryotic lineages (Apusozoa, Rhizaria, Haptophyta, and Cryptophyta) so that all the major eukaryotic supergroups are represented (Roger and Simpson 2009). Evolutionary analyses have consistently demonstrated that the evolution of parasitic phenotypes is often accompanied by large-scale gene losses (Peyretaillade et al. 2011; Pomberta et al. 2012; Wolf et al. 2013). To overcome this problem, we here include free-living representatives of lineages that were previously represented only by parasitic taxa (such as *Ectocarpus siliculosus* and unicellular brown algae in Heterokonta/Stramenopiles and *Naegleria gruberi* in Excavata). Furthermore, we include data from taxa occupying phylogenetic positions that are key to understand major evolutionary transitions, including deep-branching fungi (the Chytridiomycota *Spizellomyces punctatus*), green algae, deeply derived plants, unicellular holozoan lineages (choanoflagellates, filastereans, and ichthyosporeans) and early-branching metazoans (ctenophores and sponges). We also use improved alignment and phylogenetic inference methods. We do not aim to infer a eukaryotic tree of life from the myosin genomic content (Richards and Cavalier-Smith 2005; Odronitz and Kollmar 2007). Convergence (Zmasek and Godzik 2012) (discussed later), gene fission (Leonard and Richards 2012), duplication, gene loss (Zmasek and Godzik 2011), and horizontal gene transfer (HGT) (Andersson et al. 2003; Andersson 2005; Marcet-Houben and Gabaldón 2010; Richards et al. 2011) are important phenomena in eukaryotes and, therefore, molecular markers such as the distribution pattern of gene orthologs need to be tested using gene phylogeny and updated as new genome sequences are released (Dutilh et al. 2007; House 2009; Shadwick and Ruiz-Trillo 2012). We based our myosin classification exclusively on phylogenetic affinity, which allowed us to identify: gene and domain loss, paralog groups, and convergent evolution of gene domain architecture. The use of updated phylogenetic methods and improved taxon representation allowed us to analyse the classification, evolutionary history, and functional diversification of myosins in new detail.

## Materials and Methods

### Taxon Sampling and Sequence Retrieval

Myosin sequences were queried in complete genome or transcriptome sequences of 62 taxa representing all known eukaryotic supergroups. Taxon sampling included 8 animals, 10 unicellular holozoans, 12 fungi, 1 apusozoan, 3 amoebozoans, 5 plants, 4 chlorophytes, 2 rhodophytes, 5 heterokonts, 5 alveolates, 1 rhizarian, 1 haptophyte, 1 cryptophyte, and 4 excavates (supplementary table S2, Supplementary Material online). The complete proteomes of all included species were analysed using Pfamscan (a HMMER search-based algorithm; Punta et al. 2012) with the default gathering threshold. Using custom Perl scripts, the resulting output files were parsed and all proteins containing a Myosin_head (PF00063) domain were extracted.

### Phylogenetic Analyses

The sequences retrieved were aligned using the Mafft L-INS-i algorithm, optimized for local sequence homology (Katoh et al. 2002, 2005). The alignment was then manually inspected and edited in Geneious. This resulted in a matrix containing 353 amino acid residues, belonging to the Myosin_head domain (as this is the only conserved domain across all myosin classes). This way we avoid as well any effect that convergently acquired protein domain architectures may have while inferring the phylogeny.

Maximum likelihood (ML) phylogenetic trees were estimated by RaxML (Stamatakis 2006) using the PROTGAMMALGI model, which uses the Le and Gascuel (LG) model of evolution (Le and Gascuel 2008) and accounts for between-site rate variation with a four category discrete gamma approximation and a proportion of invariable sites (LG + Γ + I). Statistical support for bipartitions was estimated by performing 1,000-bootstrap replicates using RaxML with the same model. Bayesian inference trees were estimated with Phylobayes 3.3 (Lartillot et al. 2009), using two parallel runs for 500,000 generations and sampling every 100 and with the LG + Γ + I model of evolution. Bayesian posterior probabilities (BPP) were used for assessing the statistical support of each bipartition.

### Concurrent Domain Analysis

The domain architecture of all retrieved sequences was inferred with Pfamscan (Punta et al. 2012), using the gathering threshold as cutoff value. Then, the number of different concurrent domains (domains encoded within the same predicted open reading frame [ORF]) was calculated for each species using custom Perl scripts (excluding the myosin head domain itself). This information was further used to build Venn diagrams of shared concurrent domains between groups, using custom Bash scripts and the website: http://bioinformatics.psb.ugent.be/webtools/Venn/ (last accessed January 29, 2014).

## Results and Discussion

### Myosin Classification

**Fig. 1.—:**
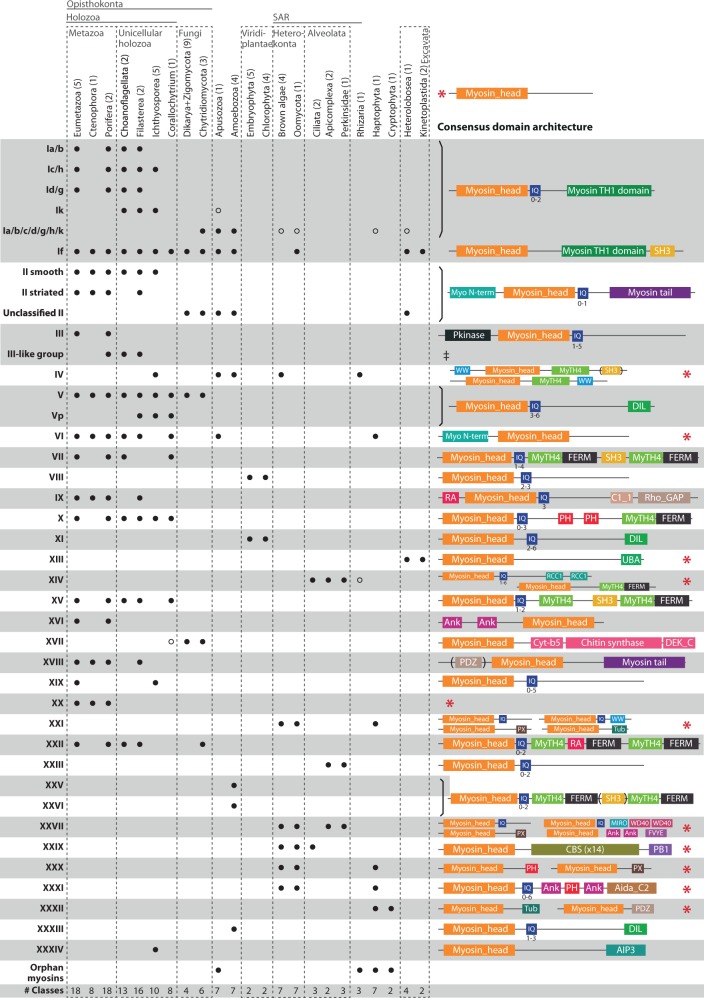
Phylogenetic distribution of myosin classes in eukaryotic genomes. The domain architectures for each class are shown, with a red asterisk on the right indicating that a single myosin head domain is also found in some sequences within that particular class. Filled circles indicate the presence of orthologs of a myosin class in a particular lineage. Unclear putative orthologs are shown with empty circles (see text). The presence of orphan myosins (i.e., species-restricted myosin classes) is also indicated. The total number of classes in each linage is indicated in the lower row. The number of species included in each taxonomic group is shown in parentheses. ‡See figure 3 for a detailed description of the various domain architectures.

Our genomic survey and phylogenetic analyses defined 31 myosin classes. Figure 1 displays their distribution across eukaryotic taxonomic groups and their canonical protein domain architecture for each class and subclass. Our data corroborated previous findings (Richards and Cavalier-Smith 2005; Foth et al. 2006; Odronitz and Kollmar 2007) and also identified a number of new families. This was somewhat expected, given that the number of myosin classes discovered has grown considerably since the pioneering studies of Cheney et al. (1993) and Goodson and Spudich (1993). For the sake of clarity, we incorporated the nomenclature used in previous studies (Cheney et al. 1993; Goodson and Spudich 1993; Hodge and Cope 2000; Berg et al. 2001; Thompson and Langford 2002; Richards and Cavalier-Smith 2005; Foth et al. 2006; Odronitz and Kollmar 2007; Syamaladevi et al. 2012), except for a number of classes in which there were conflicting names (see table S1, Supplementary Material online, for a comparison of nomenclature among studies). We dismissed and/or reused class names only on those cases in which we unambiguously inferred a different phylogenetic relationship, and therefore alternative classification, to that identified in previous analyses. Thus, our new updated and integrative classification provides a useful systematic framework for myosins.

#### Myosin I, the Largest Myosin Class, Has Five Subclasses

**Fig. 2.—:**
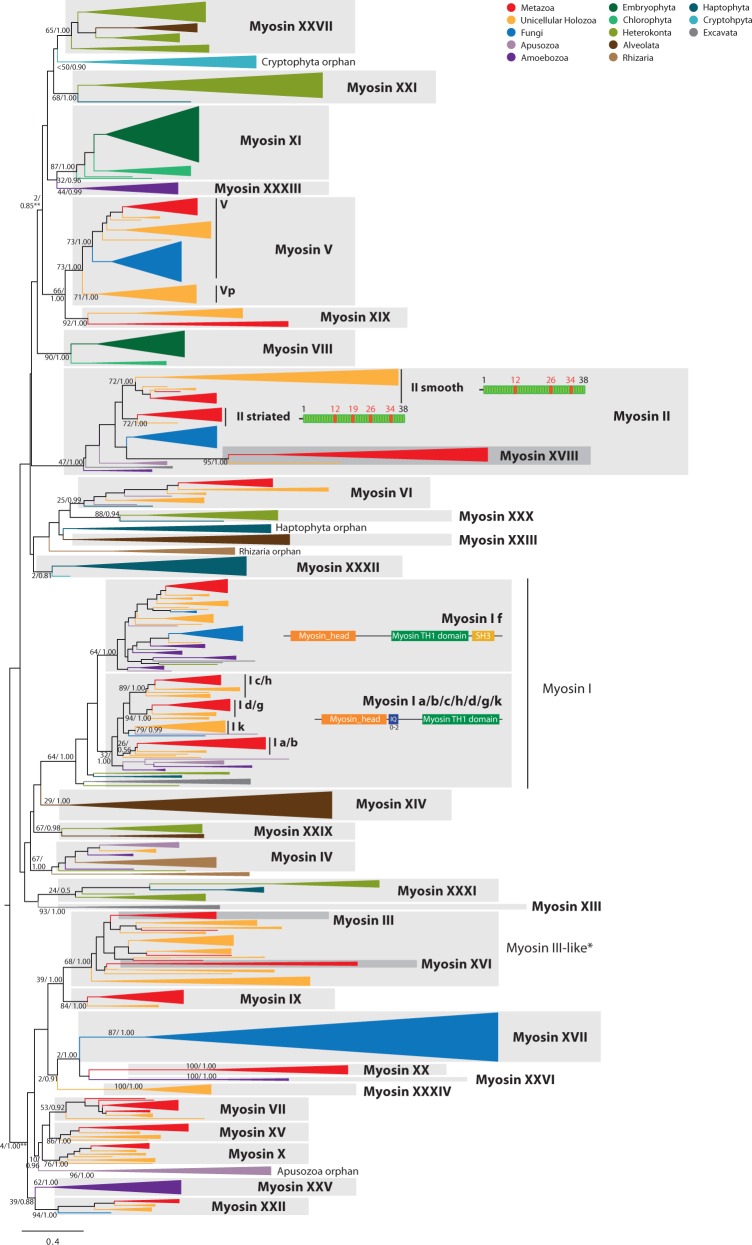
ML tree of myosin head domains. The tree is collapsed at key nodes and rooted using the midpoint-rooted tree option. Myosin classes are indicated. Nodal support was obtained using RAxML with 1,000 bootstrap replicates and BPP. Both values are shown on key branches. Taxa are color-coded according to taxonomic assignment (indicated in the upper right). The abbreviations are indicated in supplementary table S1, Supplementary Material online. Specific domain architectures are highlighted for myosin classes I and II (see text). **See figure 3 and supplementary figure S3 (Supplementary Material online) for more detail on the phylogeny of MyTH4-FERM and V-like myosins, respectively.

Myosin I (bootstrap support [BS] = 64%, BPP = 1.0; see fig. 2 and supplementary fig. S1, Supplementary Material online) comprises five subclasses including myosin Ik, newly identified here (BS = 79%, BPP = 0.99). Subclasses c/h, d/g, and a/b (named according to their vertebrate co-orthologs) have a tail composed of IQ domains (PF00612) and a myosin TH1 domain (PF06017). Co-orthologs of these four subclasses are present in several eukaryotic taxa (fig. 1). Interestingly, we find orthologs of each subclass in unicellular holozoans. Myosin Ik, which is found in choanoflagellates, filastereans, ichthyosporeans, and, with weaker support, in *Thecamonas trahens*, was lost in metazoans, and thus the diversification of these four subclasses (Ia/b, Ic/h, Id/g, and Ik) most likely occurred in the common ancestor of Holozoa prior to the radiation of Metazoa.

#### Myosin II Is Not a Valid Molecular Synapomorphy for Amorphea

Myosin II is the second largest class of myosins, and is characterized by a myosin N-terminal domain (PF02736) and a tail containing an IQ domain and a myosin tail domain (PF01576), consisting of several coiled-coil domains. Although myosin II was previously thought to be exclusive to amorpheans (also known as unikonts [Adl et al. 2012]) and was used as a phylogenetic marker (Richards and Cavalier-Smith 2005), a myosin II homolog was recently identified in the excavate *N. gruberi* (Odronitz and Kollmar 2007; Fritz-Laylin et al. 2010). Myosin II therefore probably had a deeper ancestry, although a HGT event from Amoebozoa to Excavata cannot be ruled out—especially considering the several cases of HGT that have recently been described between Heterolobosea and Amoebozoa (Andersson 2011). However, myosin proteins form numerous and specific interactions with actin filaments, plasma membrane, and numerous secondary protein complexes. Proteins with complex protein–protein interaction networks have been shown to be less likely to undergo HGT probably because integration into foreign protein interactions is limited (Jain et al. 1999; Cohen et al. 2011). Therefore, our favoured explanation for aberrant taxon distribution of myosin orthologs and domain architecture patterns identified in this study (as in the case of myosin VI discussed below) are patterns of multiple secondary loss or convergence, rather than HGT. Irrespective of whether the *N. gruberi* myosin II is a result of HGT or not, this shows that myosin II is no longer a valid molecular synapomorphy for amorpheans.

#### Striated Muscle Myosin II in Holozoa

Interestingly, myosin II is the major motor protein involved in actomyosin contraction in metazoan muscle and nonmuscle cells (Clark et al. 2007), providing contractile force during cytokinesis in the latter (Matsumura 2005), a function also performed by members of yeast myosin class II (East and Mulvihill 2011). Metazoans have two subclasses of myosin II, referred to here as smooth (Myo2) and striated (Myo11/zipper) muscle myosins (fig. 1), which have been shown to have architectural differences in the composition of their coiled-coil domains and to have originated most likely at the stem of Holozoa, although striated muscle myosin was later lost in unicellular holozoans (Steinmetz et al. 2012). We confirm this hypothesis by showing that an extant filasterean species, *Ministeria vibrans*, has a striated myosin homolog (BS = 72%, BPP = 1.0) with the extra 29 aa-based coiled-coil that is typical of striated muscle myosin II (fig. 2) (Steinmetz et al. 2012). We therefore infer that myosin II was derived early in the radiation of the eukaryotes and diverged into two classes in the holozoan lineage (smooth and striated), the latter being secondarily lost in ichthyosporeans and choanoflagellates.

#### Myosin III-Like: An Expanded Holozoan Clade

**Fig. 3.—:**
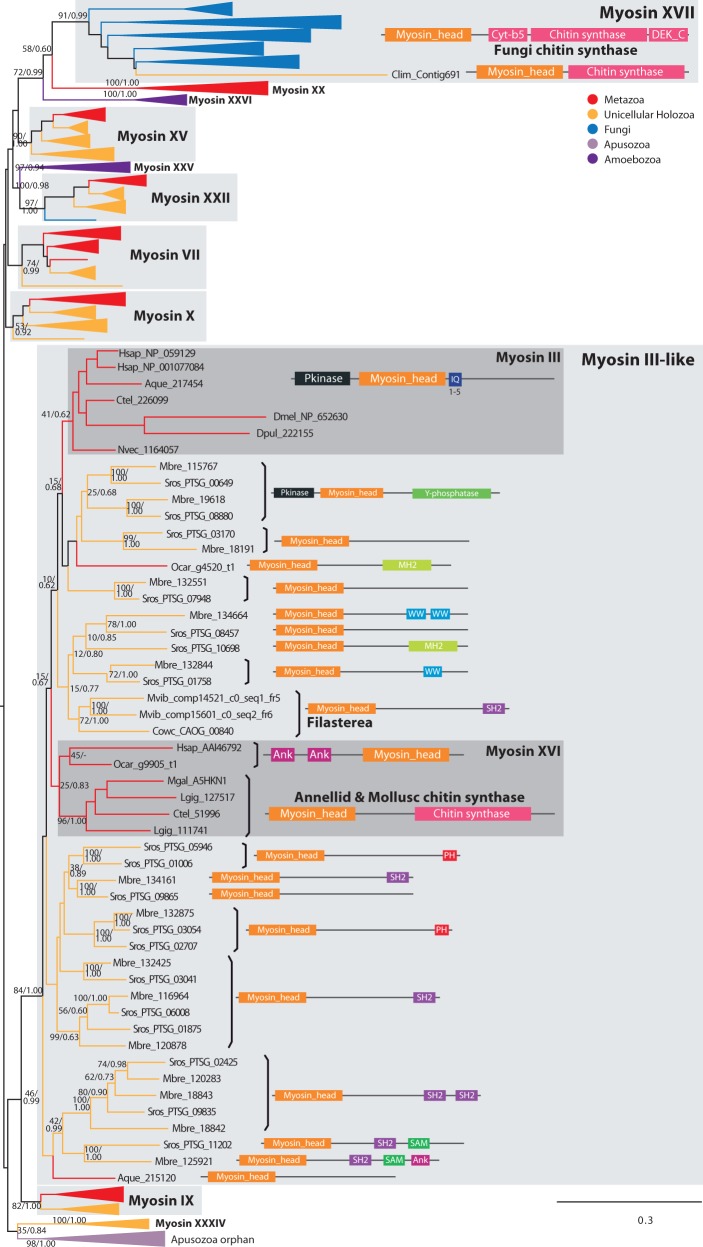
Convergent evolution of animal-fungal chitin synthases and myosin III lineage-specific expansion. ML tree of MyTH4-FERM myosin head domains. The tree is collapsed at key nodes and rooted using the midpoint-rooted tree option. Myosin classes are indicated. Statistical support was obtained by RAxML with 1,000 bootstrap replicates and BPP. Both values are shown on key branches. Taxa are color-coded according to taxonomic assignment as in figure 2. The protein domain architectures of key sequences are shown. The abbreviations are provided in supplementary table S1, Supplementary Material online.

The myosin III class is characterized by an N-terminal Protein kinase domain (PF00069) and several IQ domains (fig. 1). It is strictly metazoan-specific, although a larger group of choanoflagellate, sponge, and filasterean sequences appear to be related to it (BS = 68%, BPP = 1.0) (figs. 1, 2, and 6). This group represents a choanoflagellate-specific expansion of myosin genes, with different domain arrangements, including some members with protein kinase domains, WW domains (PF00397), SH2 domains (PF00017), PH domains (PF00169), Y-phosphatase domains (PF00102), and others (discussed later; fig. 3). The metazoan-specific myosin XVI is also related to myosin III and myosin III-like sequences. Our data demonstrate that myosin III-like originated at the stem of the Filozoa clade (i.e., Filasterea, Choanoflagellata, and Metazoa), acquiring its definitive domain configuration (with an N-terminal protein kinase domain) and leading to the birth of an additional paralog class (myosin XVI) at the base of the Metazoa.

#### Myosin IV Is Not an Orphan Acanthamoeba castellanii Myosin

**Fig. 4.—:**
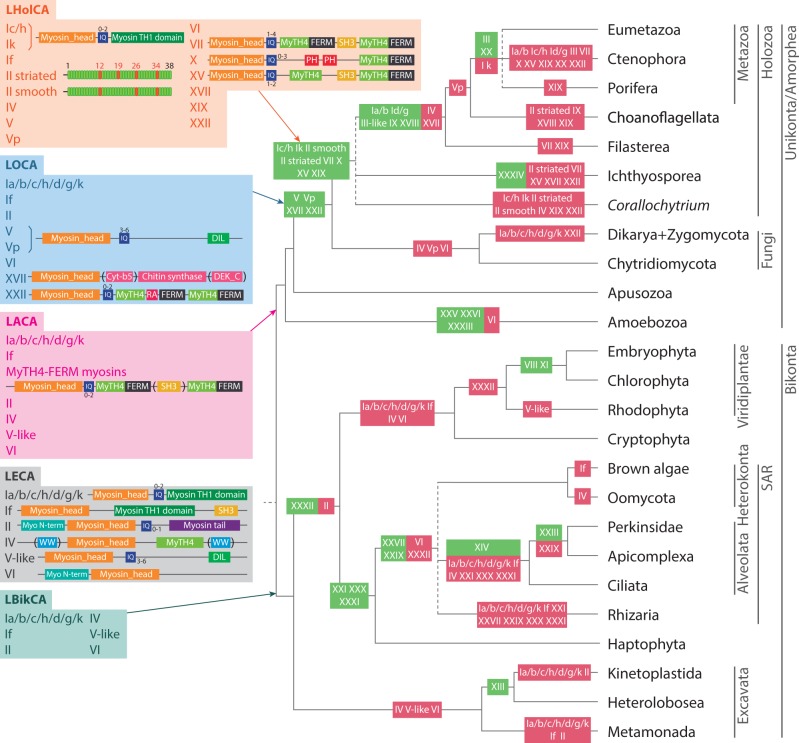
Reconstruction of myosin evolution in eukaryotes. Key ancestral nodes, including inferred domain architectures, are reconstructed, including LECA, LBikCA, LACA, modified after Derelle and Lang (2012), LOCA, and LHolCA. Domain architecture is only shown at the most ancient inferred presence of a particular myosin type (e.g., myosin if only at the LECA reconstruction). The appearance and loss of myosin classes are mapped in green and red, respectively. Dashed lines indicate unresolved phylogenetic relationships. Tree topology is based on different recent phylogenomic studies (Dunn et al. 2008; Hampl et al. 2009; Burki et al. 2012; Derelle and Lang 2012; Torruella et al. 2012; Laurin-Lemay et al. 2012; Sierra et al. 2013).

All myosin IV proteins have WW domains that can either be N-terminal or C-terminal to the Myosin_head domain, and a tail with a MyTH4 domain (PF00784), followed in some cases by a SH3 domain (in *T. trahens* and ichthyosporeans) (fig. 1). Previously considered an orphan myosin of the amoebozoan *Acanthamoeba castellanii* (Odronitz and Kollmar 2007), our results show that many other lineages have class IV myosins namely, ichthyosporeans, apusozoans, rhizarians, and heterokonts (BS = 67%, BPP = 1.0; figs. 1 and 2). Thus, despite its patchy distribution, it is likely that this myosin class was present in the LECA (fig. 4).

#### Myosin V and Related Myosins: A Large Assembly of Related Proteins

Class V myosins have an N-terminal Myosin_head domain and a C-terminal tail with IQ and a globular DIL domains (PF01843) (fig. 1). Myosin V and the structurally similar plant myosin XI carry a remarkable variety of cargo, including organelles, vesicles, and protein complexes (Li and Nebenführ 2008; Loubéry and Coudrier 2008). A relationship between myosin V and plant myosin XI has long been proposed due to their similar domain architectures (Richards and Cavalier-Smith 2005; Li and Nebenführ 2008). Moreover, the orthology between opisthokont myosin V and amoebozoan myosin V (renamed here as myosin XXXIII) was assumed but not well-supported phylogenetically (Foth et al. 2006; Odronitz and Kollmar 2007). Here, we show that all myosin V-like proteins cluster together phylogenetically with low ML nodal support in the global analysis (BS = 2%, BPP = 0.85), but maximum nodal support (BS = 100%, BPP = 1.00) if a closer outgroup is used (supplementary fig. S3, Supplementary Material online). This group includes other bikont myosins with different domain architectures. Therefore, we propose a unique ancestral origin in the LECA for the progenitor of this paralogous family (fig. 2; supplementary figs. S1–S3, Supplementary Material online). We group them in several classes, including plant myosin XI (BS = 87%, BPP = 1.0), opisthokont myosin V (BS = 73%, BPP = 1.0), amoebozoan myosin XXXIII (BS = 44%, BPP = 0.99) (formerly called myosin V, but phylogenetically not related to it), stramenopile + haptophyte myosin XXI (BS = 68%, BPP = 1.0), stramenopile + alveolate myosin XXVII (BS = 65%, BPP = 1.0), and a group of *Guillardia theta* orphan myosins (BS = 38%, BPP = 0.9) (these last three do not have the consensus myosin V architecture, presenting a wide variety of alternative domain architectures) (fig. 1). In the case of opisthokont myosin V, we confirm that myosin XIX is related to it (BS = 66%, BPP = 1.0), but we demonstrate that it is not a metazoan-specific class because it is also present in ichthyosporeans. Moreover, our phylogenetic trees strongly suggest that myosins V and Vp originated in the last common ancestor of opisthokonts (BS = 73%, BPP = 1.0) (supplementary fig. S3, Supplementary Material online). Myosin Vp was secondarily lost in fungi, metazoans, and choanoflagellates. Interestingly, the two filasterean species analysed have differentially lost one or the other, as *Capsaspora owczarzaki* has myosin Vp and *M. vibrans* has myosin V (fig. 1).

#### Myosin VI Is Mostly Specific to Opisthokonta and Apusozoa

The unique class VI myosins move toward the minus end of actin filaments, in contrast to all other known myosins. Myosins from this class are involved in diverse processes such as cytokinesis, transcription regulation, and endocytosis (Roberts et al. 2004; Sweeney and Houdusse 2010). Our phylogeny shows that homologs of this class are present in metazoans, choanoflagellates, filastereans, *Corallochytrium limacisporum*, and apusozoans, but not in fungi or amoebozoans (fig. 1). Foth et al. (2006) found putative VI-like genes in alveolates, but our analysis places them within myosin XXIII (supplementary table S1, Supplementary Material online). Yet, we identified an ortholog in the haptophyte *Emiliania huxleyi* (BS = 25%, BPP = 0.99). It is not clear whether this nonamorphean myosin VI represents an ancestral member that was lost in all other bikonts, or whether it derives from a HGT event. The fact that this and a *T. trahens* homolog share a unique C-terminal RUN domain (PF02759) that is not found in any other myosin supports the latter possibility.

#### Myosins VII, IX, X, XV, XVIII, and XIX Are Holozoan Specific

Myosins VII, IX, X, XV, XVIII, and XIX were previously considered to be unique to animals (Odronitz and Kollmar 2007), but we demonstrate the presence of clear orthologs in unicellular holozoans as well. In mammals, myosin VII is a MyTH4-FERM myosin class found in structures based on highly ordered actin filaments, such as stereocilia and microvilli (Henn and De La Cruz 2005). Its members have a tail with two MyTH4 domains (PF00784), two FERM (PF00373) domains, likely the product of a partial gene tandem duplication, and addition of a SH3 domain. Myosin VII homologs are found only in metazoans, choanoflagellates and *Co. limacisporum* (fig. 1). Some authors described a group of amoebozoan proteins with a similar architecture, involved in chemotaxis and cell polarization (Breshears et al. 2010), and identified them as VII myosins. Yet, our phylogenetic analysis does not place them with the Holozoan VII class and, therefore, we reclassify them as myosin XXV (discussed later).

Myosin VII is phylogenetically related to myosins X and XV (the other MyTH4-FERM myosins found in metazoans, discussed later) and to a group of apusozoan orphan myosins, although with low nodal support in ML analysis (BS = 10%, BPP = 0.96) (fig. 2; supplementary figs. S1 and S2, Supplementary Material online). Our results therefore suggest that all three originated from a single ancestral protein in the last common ancestor of Holozoa (being differentially lost in some unicellular lineages; only the unicellular *Co. limacisporum* has orthologs of all three classes, XV, X, and VII). Interestingly, ctenophores have lost these three myosin classes. Myosin IX is composed of a N-terminal RA domain (PF00788) and a tail with IQ domains, a C1_1 domain (PF00130) and a RhoGAP domain (PF00620). Homologs of this class are found only in metazoans and filasterea (fig. 1).

Myosin X and XV are MyTH4-FERM classes of crucial importance for metazoan filopodia (Zhang et al. 2004; Bohil et al. 2006; Liu et al. 2008). The tail of myosins X is composed of a variable number of IQ motifs, two PH (PF00169), one MyTH4, and one FERM domain; while those of myosins XV are composed of two MyTH4, one FERM, and one SH3 domain. Myosin XVIII often has an N-terminal PDZ domain and has a C-terminal myosin tail domain. This family is present in the filasterean *C. owczarzaki* and all metazoans examined (BS = 95%, BPP = 1.0) (fig. 1). Although not statistically supported, myosin XVIII could be closely related to myosin II, as previously described (Foth et al. 2006). Finally, myosin XIX has a variable number of IQ domains and it is only found in eumetazoans and ichthyosporeans (BS = 92%, BPP = 1.0) (fig. 1). It is closely related to myosin V (BS = 66%, BPP = 1.0) (fig. 2; supplementary figs. S1–S3, Supplementary Material online).

#### Myosin VIII and XI: The Green Lineage Myosins

Myosins VIII and XI are the only myosin classes present in plants and several chlorophytes (Peremyslov et al. 2011; fig. 1). Myosin VIII, whose monophyly is strongly supported (BS = 90%, BPP = 1.0), has a tail with IQ domains. As for myosin XI, several authors have pointed out its strong similarity to myosin class V in terms of domain architecture (Thompson and Langford 2002; Foth et al. 2006; Li and Nebenführ 2008). Here, we show that this class is found in embryophytes and chlorophytes and is well supported (BS = 87%, BPP = 1.0; fig. 1). This class is phylogenetically related to myosin V, and is included in a major myosin cluster that we name myosin V-like (fig. 2; supplementary figs. S1–S3, Supplementary Material online).

#### Myosin XIV: Myosins with a MyTH4-FERM Protein Domain Combination in a Ciliate

Myosin XIV has been shown to be involved in phagosome motility and nuclear elongation in the ciliate *Tetrahymena thermophila* (Williams and Gavin 2005; Foth et al. 2006). We find that this is an alveolate-specific class that has expanded in many species (specifically in ciliates) and that shows various domain architectures. Interestingly, the ciliate *Te. thermophila* has several myosin XIV homologs with MyTH4 and FERM domains, and is the only known bikont (nonamorphean) taxon with myosins that have a MyTH4-FERM protein domain combination. This configuration is very common in amorphean myosins, and was probably convergently acquired in the ciliates.

#### Myosin XVI and XVII: Convergence of Fungal and Animal Myosins with a C-terminal Chitin Synthase

Myosin XVII, also called chitin synthase, is a fungus transmembrane myosin with Cyt-b5 (PF00173), chitin synthase 2 (PF03142) and DEK_C (PF08766) domains in its tail, a domain combination unique to this class. Its monophyly is well supported (BS = 91%, BPP = 0.99), and it is phylogenetically related to amorphean FERM domain myosins. This chitin synthase class was thought to be specific to Fungi (James and Berbee 2012). Interestingly, the holozoan *Co. limacisporum* has a highly derived myosin that is associated with a chitin synthase domain and that is phylogenetically related to the fungal myosin XVII (fig. 3). This implies that class XVII chitin synthase precedes the appearance of the Opisthokonta and was lost in most holozoan lineages (except for *Co. limacisporum*) and so is not a valid synapomorphy for the fungi (James and Berbee 2012). Moreover, we also identified myosins with chitin synthases in annelids and molluscs (figs. 1 and 3), which are members of the XVI class. Thus, they are not orthologous to fungus chitin synthases, but rather appeared convergently in annelids and molluscs (fig. 3).

#### Myosin XXII: An Opisthokont-Specific Myosin with a Scattered Taxonomic Distribution

Myosin XXII is a MyTH4-FERM domain myosin found in some opisthokonts, including the chytrid fungus *S. punctatus*, filastereans, choanoflagellates, poriferans, and *Drosophila melanogaster*. Its tail is composed of an IQ, two MyTH4 and two FERM domains, with a RA domain (PF00788) between the first MyTH4 and the first FERM domain. It was secondarily lost in *Co. limacisporum*, ichthyosporeans, and many metazoans (fig. 1). Myosin XXII seems to be related to amoebozoan myosin XXV (fig. 2). They may comprise a single class, although there are some architectural differences between them (discussed later).

#### Myosin XXI, XXX, and XXXI: Heterokonta and Haptophyta Share Unique Myosins

These three myosin classes are found in heterokonts and haptophytes, which suggests that they were secondarily lost in rhizarians and alveolates (figs. 1 and 4) as these groups are thought to branch closer to heterokonts than haptophytes (Burki et al. 2012). Myosin XXI homologs present diverse myosin tail architectures, including IQ, WW (PF00397), PX (PF00787), and Tub (PF01167) domains. This class has become considerably expanded in the oomycete *Phytophthora infestans*. Myosin XXX homologs in *E. siliculosus* have a C-terminal PH domain and *P. infestans* homologs have a PX domain. Finally, the myosin XXXI class, in which we also include the old myosin XXXIII (Odronitz and Kollmar 2007), has a characteristic tail architecture in several heterokonts homologs, with a variable number of IQ domains, a PH domain flanked by two ankyrin domains, and a C-terminal Aida_C2 domain (PF14186).

#### Myosin XXV, XXVI, and XXXIII: Renamed Amoebozoa-Specific Myosins

The myosin XXV class (BS = 62%, BPP = 1.0) comprises amoebozoan sequences that were previously considered to be myosin VII homologs. They are MyTH4-FERM myosins known to have a role in cell adhesion and filopodia formation (Breshears et al. 2010). They show remarkable architectural similarities with both myosin XV and myosin VII (fig. 1), but seem to be phylogenetically related to myosin XXII (although they have different tail architectures and their sister-group relationship is low supported) (fig. 2; supplementary figs. S1 and S2, Supplementary Material online), and thus were classified as an independent class. Myosin XXVI (BS = 100%, BPP = 1.0) is another class of amoebozoan MyTH4-FERM myosins, which does not cluster with either myosin VII or myosin XXV. We suggest a common ancestry for a group of amorphean myosin classes that are generally characterised by the presence of MyTH4 domains. This group includes these two amoebozoan classes (XXV and XXVI; fig. 2; supplementary figs. S1, S2, and S4, Supplementary Material online), as well as myosins III, XVI, IX, XVII, XX, XXXIV, X, XV, VII, and XXII (fig. 3; supplementary fig. S4, Supplementary Material online).

Myosin XXXIII includes the amoebozoan sequences previously considered as class V myosin, and shares the same domain architecture as plant myosin XI. Our phylogenetic analysis does not support a close relationship between myosin XXXIII and myosin V; it rather demonstrates that they are related to the myosin V-like clade (fig. 2), leading us to rename the group as myosin XXXIII (fig. 2; supplementary figs. S1–S3, Supplementary Material online).

### The Evolution of the Myosin Repertoire in Eukaryotic Genomes

Phylogenetic analysis allowed us to define broader groups of myosin classes and to reconstruct the evolution of the myosin toolkit across the eukaryotes. This reconstruction is based on the favored hypothesis for the root of eukaryotes, the unikont–bikont split (Stechmann and Cavalier-Smith 2002; Richards and Cavalier-Smith 2005), that has recently been recovered in a rooted multi gene concatenated phylogeny with a modification with regards to the placement of the apusozoan *T. trahens* within the unikonts (Derelle and Lang 2012). Based on this root, our data suggest that the LECA had at least six myosin types, with different protein domain architectures (fig. 4 for the reconstruction of LECA and other ancestral nodes). According to our reconstruction, LECA had the following: 1) an ancestral myosin I (progenitor paralog of the myosin I a/b/c/h/d/g/k ortholog subfamilies) with an architecture consisting of a myosin head domain followed by 0 to 2 IQ repeats and a C-terminal myosin TH1 domain; 2) a myosin If, with a myosin head domain followed by a myosin TH1 domain and a C-terminal SH3 domain; 3) a myosin II, with a myosin N-terminal domain, a myosin motor domain, 0 to 1 IQ domains and a myosin tail domain; 4) a myosin IV with a myosin head domain followed by a MyTH4 domain and a characteristic WW domain (either C-terminal or N-terminal); 5) a myosin V-like myosin with a myosin head followed by variable number of IQ repeats and a C-terminal DIL domain; and 6) a myosin VI, with a myosin N-terminal domain followed by a myosin head domain.

In figure 4, we show the diversity of the myosin complement in the LECA genome under a modified version of the unikont–bikont root. Our reconstruction indicates that LECA possessed a minimum of six paralog families all encoding different protein domain architectures. Even if alternative rooting hypothesis are taken into account (Rodríguez-Ezpeleta et al. 2007; Wideman et al. 2013) the inferred number of myosin paralog families in the LECA is still high (supplementary figs. S5 and S6, Supplementary Material online). This result is consistent with the pattern observed in the kinesin gene family, which also demonstrated a diverse repertoire of paralog families present in the LECA (Wickstead et al. 2010). Together these data suggest that the LECA possessed a complex and diversified actin and tubulin cytoskeleton and that this ancestral cell possessed a large number of complex eukaryotic cellular characteristics prior to the diversification of extant and sampled eukaryotic groups. Assuming this root, these results have two implications: 1) they strongly suggest that a large quantity of protein diversification and cellular complexity evolved between the point of eukaryogenesis (Martin et al. 2001) and LECA, and 2) indicate that gene loss and subsequent reduction in cytoskeletal systems played a significant role in the diversification of eukaryotes, a pattern that is increasingly apparent on other gene families and cellular systems (Wolf and Koonin 2013).

**Fig. 5.—:**
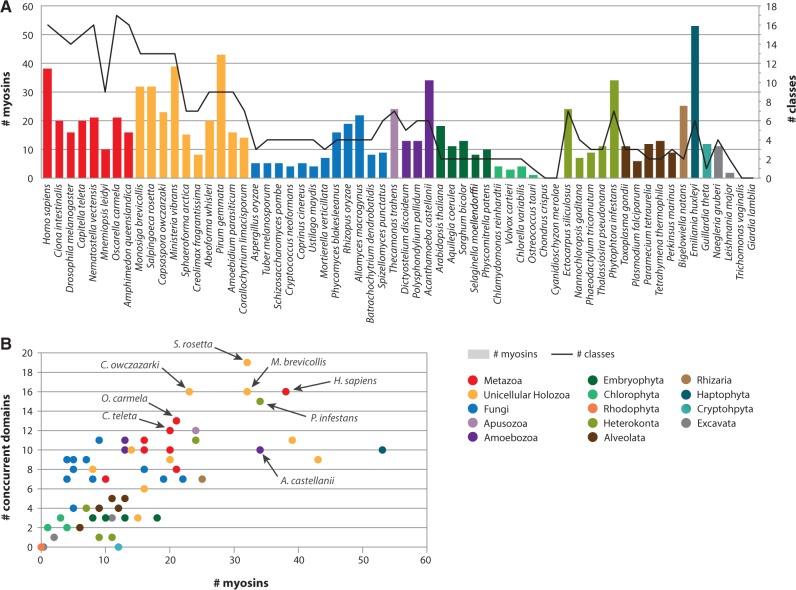
Phylogenetic patterns of myosin diversity. Taxa are color-coded according to taxonomic assignment. (*A*) Number of myosin genes (right *Y*-axis, columns) and number of myosin classes (left *Y*-axis, black line) in each species. (*B*) Number of concurrent protein domains (*Y*-axis) compared with the number of myosin genes (*X*-axis) in each species.

Our analysis reconstructed the LBikCA (Last Bikont Common Ancestor) with the same complement of myosins as the LECA (fig. 4). New classes appeared later in bikont evolution, such as myosin XIII at the stem of Kinetoplastida + Heterolobosea and myosin XXI, XXX, and XXXI at the stem of SAR + Haptophyta. Assuming the unikont–bikont root, our analyses demonstrate that many groups underwent secondary losses, with two extreme cases of complete loss of the myosin toolkit in the following: 1) metamonads (including *Trichomonas vaginalis* and *Giardia lamblia*) and 2) rhodophytes (including the unicellular *Cyanidioschyzon merolae* and the multicellular alga *Chondrus crispus*) (figs. 4 and 5).

The LACA (Last Amorphean Common Ancestor, modified by inclusion of Apusozoa [Derelle and Lang 2012]) added a new myosin type from LECA, a MyTH4-FERM myosin (Berg 2001; Richards and Cavalier-Smith 2005) that includes several phylogenetically related myosin classes (supplementary figs. S1, S2, and S4, Supplementary Material online). These myosins have a complex protein domain architecture including a myosin head domain followed by 0 to 2 IQ repeats, a MyTH4 domain, a FERM domain, in some cases a SH3 domain, and an additional MyTH4 and FERM domains (fig. 4). This ancestral protein domain architecture is found in diverse myosins from extant amoebozoans (classes XXV and XXVI) to holozoans (class VII and, with some variations, classes XV and X). In any case, the putative ancestral MyTH4-FERM myosin underwent major architectural rearrangements as the family expanded during diversification of the amorpheans (figs. 3 and 4).

The LOCA (Last Opisthokont Common Ancestor) had an even more complex myosin complement, with the addition of new myosin classes (as a consequence of the diversification of ancestral myosin types, such as myosin V-like or MyTH4-FERM myosins), including myosin V, myosin Vp, myosin XVII (a chitin synthase that is present in all fungi and in a single holozoan species, *Co. limacisporum*, discussed earlier), and myosin XXII. This complexity became even greater in the LHolCA (Last Holozoan Common Ancestor), which had the highest diversity of myosin types among all reconstructed ancestors (fig. 4). This diversity was further expanded during holozoan evolution, with little innovation at the stem of Metazoa.

### Phylogenetic Patterns of Myosin Diversity and Protein Domain Combinations

Our data show that there are strong phylogenetic patterns across lineages, in terms of abundance and number of classes, and the diversity of concurrent domains (i.e., domains that appear together with the myosin head domain in a given protein or ORF).

The number of myosin genes varies markedly between lineages (fig. 5*A*). Holozoan genomes, as well as some amoebozoans and heterokonts, have the highest numbers of myosins of all eukaryotes. In particular, the haptophyte *Em. huxleyi* has the highest number of myosin genes (53), followed by the ichthyosporean *Pirum gemmata* (43), the filasterean *M. vibrans* (39), and the metazoan *Homo sapiens* (38). On the other hand, dikaryan fungi, plants, green algae, alveolates, and some excavates have few or no myosins.

A comparison of the abundance of myosin proteins with the diversity of myosin classes (fig. 5*A*), reveals that *Em. huxleyi*, which has a high number of myosins, has only six myosin classes. This implies that the high number of myosin homologs found in this species is due to class-specific expansions rather than possession of a wide diversity of ancestrally derived myosin types. In contrast, many unicellular holozoans, especially choanoflagellates and filastereans, and some metazoans (such as *H. sapiens* and the homoscleropmorph sponge *Oscarella carmela*) have a high diversity of myosin classes. In general, our data reveal a marked increase in the number of myosin classes at the origin of Holozoa, although some specific taxa, such as the ctenophore *Mnemiopsis leidyi* and the ichthyosporeans *Sphaeroforma arctica* and *Creolimax fragrantissima*, secondarily reduced their repertoire of myosins.

Myosin motor domains are found in a diverse collection of protein domain architectures, therefore another aspect that reflects differences in myosin diversity is the number of concurrent protein domains found associated with the motor domain (fig. 5*B*). The richest species in terms of protein domain diversity attached to the myosin motor domain within a putative ORF are the choanoflagellate *Salpingoeca rosetta*, the filastereans *M. vibrans* and *C. owczarzaki* and the metazoan *H. sapiens*. This implies that myosins were highly diversified prior to the origin and divergence of metazoans. Indeed, the sponge *O. carmela* also has a rich repertoire of concurrent domains, which corroborates (together with the fact that it has the richest range of myosin classes among analysed taxa) that the myosin repertoire was already rich and diverse in early metazoan evolution.

Interestingly, the oomycete plant pathogen *P. infestans*, which has a high number of myosin genes, also shows a remarkable diversity of concurrent protein domains (Richards and Cavalier-Smith 2005), a feature that has already been described for other gene families (Grau-Bové et al. 2013). In contrast, the myosin-rich taxon *Em. huxleyi* is relatively poor in both class diversity (fig. 5*A*) and protein domain diversity. The poorest taxa in protein domain diversity are plants, chlorophytes, excavates and alveolates. The cryptophyte *G. theta* represents an extreme case with no identified protein domains within the predicted ORF of any of its 11 myosins.

**Fig. 6.—:**
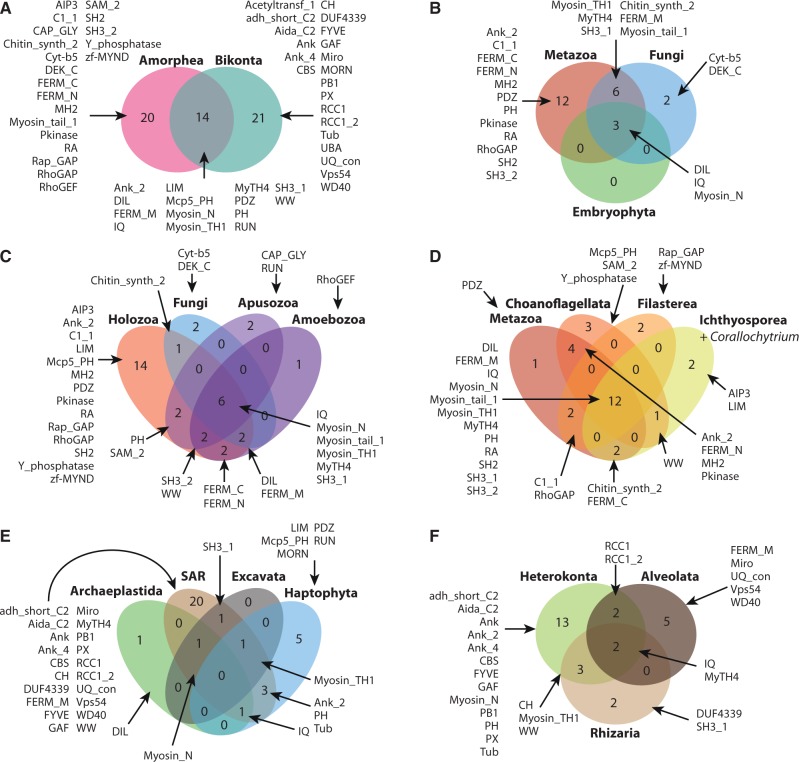
Myosin concurrent protein domains in different eukaryotic lineages. Venn diagrams show the number of shared and lineage-specific protein domains in different comparisons. The Pfam names of the various protein domains are indicated.

An examination at the concurrent protein domain composition of myosin in different taxa (fig. 6) reveals that 14 protein domains are conserved between amorpheans and bikonts (fig. 6*A*) with similar levels of innovation in both clades (20 and 21 new concurrent protein domains, respectively). A comparison of the most widely studied eukaryote clades (metazoans, embryophytes, and fungi [fig. 6*B*]) reveals that there are no specific concurrent domains in plants (only those present in myosin XI, which are shared by metazoan and fungus myosin class V) and in fungi there are only two specific domains (those associated with myosin XVII, i.e., DEK_C and Cyt-b5). In contrast, metazoans have many specific domains associated with myosins.

Within amorpheans (fig. 6*C*) there is a core of conserved domains (such as Myosin_tail_1 or Myosin_TH1) and a burst of innovation in the Holozoa. A closer look reveals that most of these domain combinations are present in unicellular holozoans, while little actual innovation occurred at the origin of metazoans (only the PDZ domain) (fig. 6*D*). In contrast, every single unicellular holozoan lineage has new specific associated domains: three in choanoflagellates (Mcp5_PH, SAM_2 and Y_phosphatase), two in filastereans (Rap_GAP and zf-MYND) and two in ichthyosporeans (AIP3 and LIM).

Within bikonts (fig. 6*E*) there are no protein domains shared by all major lineages and little innovation in protein domain combinations is observed, except in the case of haptophytes (five domains) and particularly in the SAR clade (Stramenopiles/Heterokonta, Alveolata, and Rhizaria). A closer look at the SAR clade (fig. 6*F*) reveals that this diversification of protein domains is largely lineage-specific, with five new domains in alveolates and thirteen new domains in heterokonts.

It is interesting to note that some of these shared protein domains were acquired convergently, for example the LIM domain in haptophytes and ichthyosporeans, the Mcp5_PH domain in haptophytes and choanoflagellates and the FERM_M domain in alveolates and amorpheans. This points to another source of homoplasy when considering protein domain architectures as evolutionary synapomorphies.

### Lineage-Specific Myosin Diversifications

Our data show several lineage-specific expansions, often accompanied by major protein domain architecture rearrangements. This is the case, for example, of myosin class XXVII, which is expanded in both the oomycete *P. infestans* and the alveolate *Perkinsus marinus*, with unique protein domain architectures. Another example is the ciliate *Te. thermophila*, which has 12 myosin homologs of the alveolate-specific class XIV. In addition to the consensus architecture found in most alveolates, *Te. thermophila* myosin XIV is the only bikont myosin with the MyTH4-FERM domain combination, a domain architecture that was convergently acquired (compared with amorphean MyTH4-FERM myosins, discussed earlier).

The most spectacular lineage-specific expansion is that observed in choanoflagellate myosin III-like myosins (fig. 3). This phylogenetically defined group includes bona fide eumetazoan myosin III homologs, the related metazoan myosin XVI class (including annelid and mollusc chitin synthases), filasterean sequences (comprising a unique group), a single sequence of the sponge *Amphimedon queenslandica*, a single sequence of the sponge *O. carmela*, and several choanoflagellate myosins (15 from *Monosiga brevicollis* and 18 from *Sa. rosetta*). These choanoflagellate sequences have a wide diversity of protein domain rearrangements (fig. 3). Interestingly, many of these domains, like SH2 and Y-phosphatase domains, are related to tyrosine kinase signaling (Liu et al. 2011), a prominent feature of choanoflagellates (Manning et al. 2008). Sequences belonging to the myosin III-like group with a C-terminal SH2 domain were also identified in filastereans, which also have an extensive tyrosine kinase toolkit (Suga et al. 2012). Another interesting configuration found within this myosin III-like group is an *Sa. rosetta* and an *O. carmela* sequence with a C-terminal MH2 PF03166 domain. This domain is typically present in Smad transcription factors, where it is found at the C-terminal of the MH1 DNA-binding domain and acts as a protein binding motif that mediates cofactor interactions (Massagué et al. 2005). Interestingly, the MH2 domain is only found in choanoflagellates and metazoans, while Smad transcription factors are exclusive to animals (Sebé-Pedrós et al. 2011). The fact that the single MH2 domain found in choanoflagellates is associated with a myosin, together with that fact that the sponge *O. carmela* also has this configuration, suggests that MH2 initially appeared associated with myosins as a protein–protein interaction domain. Later on, early in metazoan evolution, MH2 was fused by domain shuffling to a MH1 DNA-binding domain to create the Smad transcription factors.

### The Origin of the Metazoan Myosin Repertoire

Our results show that all metazoan myosin classes but one (Myosin XVI, also known as Dachs) have a premetazoan origin, many of them being holozoan innovations (fig. 6) (including myosin III-like, VII, IX, X, XV, XVIII, and XIX). Moreover, several subclass diversifications occurred in unicellular holozoans, for example in Myosin V (Myosin V and Myosin Vp), in Myosin I (Myosin I a/b, I/c/h, Id/g, and Ik) and in Myosin II (smooth and striated). In terms of number of myosins and diversity of concurrent domains (fig. 5), unicellular Holozoa have the highest counts among eukaryotes (even higher than most Metazoa). In fact, the choanoflagellate *Sa. rosetta* has the most diverse repertoire of myosin concurrent domains (fig. 5*B*), followed by another choanoflagellate (*Mo. brevicollis*), the filasterean *C. owczarzaki* and the metazoan *H. sapiens.* Overall, we can infer that the complexity of the myosin toolkit was extremely high before the advent of animal multicellularity and that this system is of paramount importance in extant unicellular holozoans.

## Conclusions

We provide a robust updated myosin classification, based on ML and Bayesian phylogenetic methods and broad genomic taxon sampling that includes, for the first time, all major eukaryotic lineages. We provide a redefinition and/or confirmation of previously defined myosin classes (with an effort to reconcile myosin nomenclature between various previous classifications), and we assess the presence/absence of myosin classes in eukaryotes. Furthermore, we reconstruct a more complex myosin complement in the LECA genome than previously proposed, with six different myosin types and six different inferred domain architectures under the modified unikont–bikont root. Notably, we find strong phylogenetic patterns related to the complexity of the myosin system. Finally, we infer an intricate evolutionary history of the myosin gene family, including multiple lineage-specific expansions (such as the myosin III-like group in the choanoflagellate lineage), domain diversifications (specially in holozoans), secondary losses (in metamonads and rhodophytes), and convergences (e.g., in the fungal and metazoan myosin–chitin synthases). Taken together our results demonstrate that myosin gene family underwent multiple large-scale expansions and contractions in paralog families combined with extensive remodelling of domain architectures. As the diversity of this gene family directly relates to the function of the actin cytoskeleton, these results tell a story of extensive remodelling of this cytoskeleton system across the eukaryotes. These results also suggest that evolutionary inference of species relationships based on myosin distribution patterns is difficult without reliable phylogenetic analysis and comprehensive sampling. As such, the expansion of available genome data will provide a more accurate inference of the relative phylogenetic age of myosin classes and types—likely expanding the repertoire of myosins, and therefore the cellular complexity, of ancestral eukaryotic forms.

## Supplementary Material

Supplementary data S1, figures S1–S6, and tables S1 and S2 are available at *Genome Biology and Evolution* online (http://www.gbe.oxfordjournals.org/).

Supplementary Data
