# Supplementary material for: Evolution and Classification of Myosins, a Paneukaryotic Whole-Genome Approach
Source: Genome Biol Evol. 2014 Jan 18;6(2):290–305. doi: 10.1093/gbe/evu013 (PMC3942036; doi:10.1093/gbe/evu013)
Supplement: Supplementary Data [file supp_6_2_290__index.html]

Evolution and Classification of Myosins, a Paneukaryotic Whole-Genome Approach — Supplementary Data 

# Evolution and Classification of Myosins, a Paneukaryotic Whole-Genome Approach

## Supplementary Data

files

**Files in this Data Supplement:**

- Supplementary Data - zip file
