# Supplementary material for: Evolution and Classification of Myosins, a Paneukaryotic Whole-Genome Approach
Source: Genome Biol Evol. 2014 Jan 18;6(2):290–305. doi: 10.1093/gbe/evu013 (PMC3942036; doi:10.1093/gbe/evu013)
Supplement: Supplementary Data [file supp_evu013_suppl_data.zip › Sebe-Pedros_myosin_SupplMaterial.pdf]

Figure S1

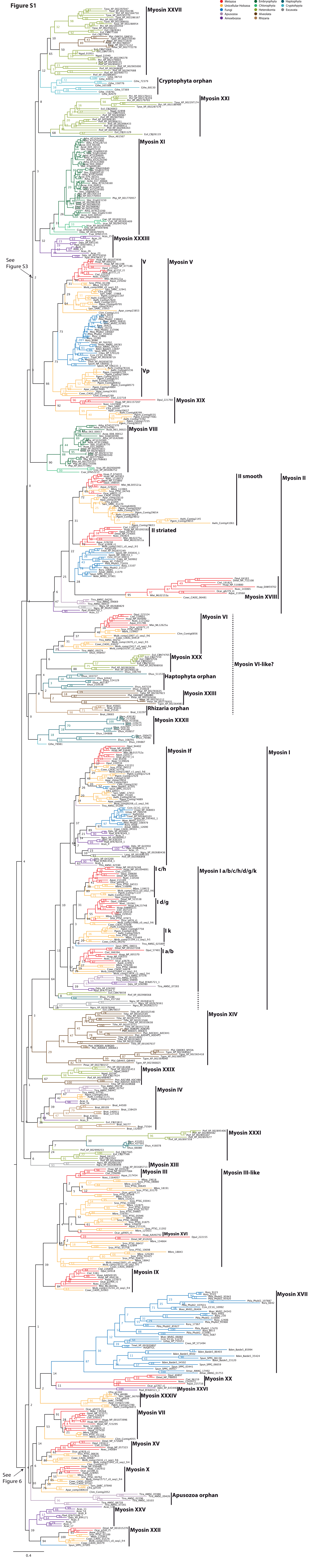

Metazoa
Unicellular Holozoa
Fungi
Apusozoa
Amoebozoa
Embryophyta
Chlorophyta
Heterokonta
Alveolata
Rhizaria
Haptophyta
Cryptophyta
Excavata
Myosin XXVII
Cryptophyta orphan
Myosin XXI
Myosin XI
Myosin XXXIII
See Figure S3
V
Myosin V
Vp
Myosin XIX
Myosin VIII
II smooth
Myosin II
II striated
Myosin XVIII
Myosin VI
Myosin XXX
Myosin VI-like?
Haptophyta orphan
Myosin XXIII
Rhizaria orphan
Myosin XXXII
Myosin If
Myosin I
I c/h
Myosin I a/b/c/h/d/g/k
I d/g
I k
I a/b
Myosin XIV
Myosin XXIX
Myosin IV
Myosin XXXI
Myosin XIII
Myosin III
Myosin III-like
Myosin XVI
Myosin IX
Myosin XVII
Myosin XX
Myosin XXVI
Myosin XXXIV
Myosin VII
Myosin XV
Myosin X
Apusozoa orphan
Myosin XXV
Myosin XXII
See Figure 6
0.4

Figure S2

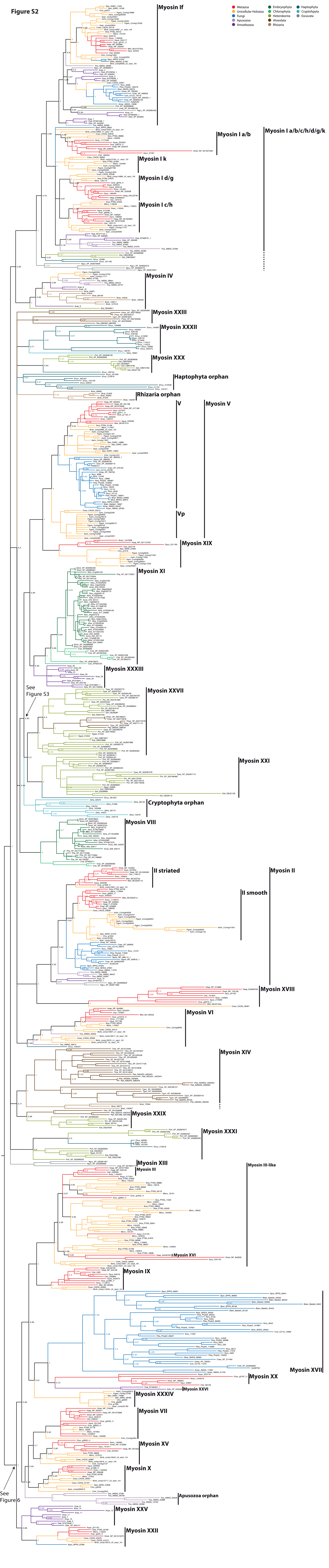

Metazoa
Unicellular Holozoa
Fungi
Apusozoa
Amoebozoa
Embryophyta
Chlorophyta
Heterokonta
Alveolata
Rhizaria
Haptophyta
Cryptophyta
Excavata
Myosin If
Myosin I a/b
Myosin I a/b/c/h/d/g/k
Myosin I k
Myosin I d/g
Myosin I c/h
Myosin IV
Myosin XXIII
Myosin XXXII
Myosin XXX
Haptophyta orphan
Rhizaria orphan
V
Myosin V
Vp
Myosin XIX
Myosin XI
Myosin XXXIII
See Figure S3
Myosin XXVII
Myosin XXI
Cryptophyta orphan
Myosin VIII
II striated
Myosin II
II smooth
Myosin XVIII
Myosin VI
Myosin XIV
Myosin XXIX
Myosin XXXI
Myosin XIII
Myosin III
Myosin III-like
Myosin XVI
Myosin IX
Myosin XVII
Myosin XX
Myosin XXXIV
Myosin XXVI
Myosin VII
Myosin XV
Myosin X
See Figure 6
Apusozoa orphan
Myosin XXV
Myosin XXII

Figure S3

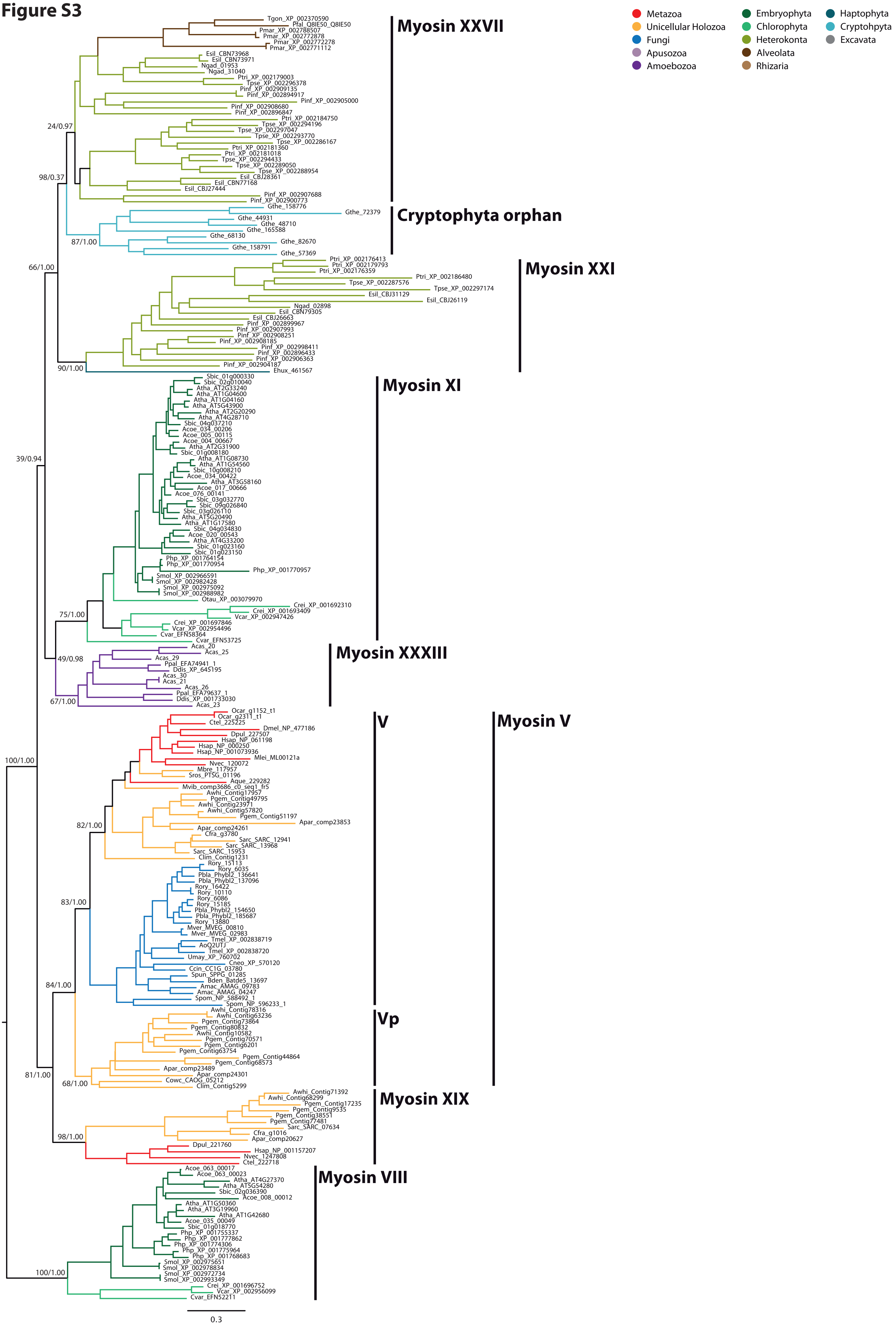

Metazoa
Unicellular Holozoa
Fungi
Apusozoa
Amoebozoa
Embryophyta
Chlorophyta
Heterokonta
Alveolata
Rhizaria
Haptophyta
Cryptohpyta
Excavata
Myosin XXVII
Cryptophyta orphan
Myosin XXI
Myosin XI
Myosin XXXIII
V
Myosin V
Vp
Myosin XIX
Myosin VIII
24/0.97
98/0.37
87/1.00
66/1.00
90/1.00
39/0.94
75/1.00
49/0.98
67/1.00
100/1.00
82/1.00
83/1.00
84/1.00
81/1.00
68/1.00
98/1.00
100/1.00
0.3
Tgon_XP_002370590
Pfal_Q8IE50_Q8IE50
Pmar_XP_002788507
Pmar_XP_002772878
Pmar_XP_002772278
Pmar_XP_002771112
Esil_CBN73968
Esil_CBN73971
Ngad_01953
Ngad_31040
Ptri_XP_002179003
Tpse_XP_002296378
Pinf_XP_002909135
Pinf_XP_002894917
Pinf_XP_002905000
Pinf_XP_002908680
Pinf_XP_002896847
Ptri_XP_002184750
Tpse_XP_002294196
Tpse_XP_002297047
Tpse_XP_002293770
Tpse_XP_002286167
Ptri_XP_002181360
Ptri_XP_002181018
Tpse_XP_002294433
Tpse_XP_002289050
Tpse_XP_002288954
Esil_CBJ28361
Esil_CBN77168
Esil_CBJ27444
Pinf_XP_002907688
Pinf_XP_002900773
Gthe_158776
Gthe_72379
Gthe_44931
Gthe_48710
Gthe_165588
Gthe_68130
Gthe_82670
Gthe_158791
Gthe_57369
Ptri_XP_002176413
Ptri_XP_002179793
Ptri_XP_002176359
Ptri_XP_002186480
Tpse_XP_002287576
Tpse_XP_002297174
Esil_CBJ31129
Esil_CBJ26119
Ngad_02898
Esil_CBN79305
Esil_CBJ26663
Pinf_XP_002899967
Pinf_XP_002907993
Pinf_XP_002908251
Pinf_XP_002908185
Pinf_XP_002998411
Pinf_XP_002896433
Pinf_XP_002906363
Pinf_XP_002904187
Ehux_461567
Sbic_01g000330
Sbic_02g010040
Atha_AT2G33240
Atha_AT1G04600
Atha_AT1G04160
Atha_AT5G43900
Atha_AT2G20290
Atha_AT4G28710
Sbic_04g037210
Acoe_034_00206
Acoe_005_00115
Acoe_004_00667
Atha_AT2G31900
Sbic_01g008180
Atha_AT1G08730
Atha_AT1G54560
Sbic_10g008210
Acoe_034_00422
Atha_AT3G58160
Acoe_017_00666
Acoe_076_00141
Sbic_03g032770
Sbic_09g026840
Sbic_03g026110
Atha_AT5G20490
Atha_AT1G17580
Sbic_04g034830
Acoe_020_00543
Atha_AT4G33200
Sbic_01g023160
Sbic_01g023150
Php_XP_001764154
Php_XP_001770954
Php_XP_001770957
Smol_XP_002966591
Smol_XP_002982428
Smol_XP_002975092
Smol_XP_002988982
Otau_XP_003079970
Crei_XP_001692310
Crei_XP_001693409
Vcar_XP_002947426
Crei_XP_001697846
Vcar_XP_002954496
Cvar_EFN58364
Cvar_EFN53725
Acas_20
Acas_25
Acas_29
Ppal_EFA74941_1
Ddis_XP_645195
Acas_30
Acas_21
Acas_26
Ppal_EFA79637_1
Ddis_XP_001733030
Acas_23
Ocar_g1152_t1
Ocar_g2311_t1
Ctel_225225
Dmel_NP_477186
Dpul_227507
Hsap_NP_061198
Hsap_NP_000250
Hsap_NP_001073936
Mlei_ML00121a
Nvec_120072
Mbre_117957
Sros_PTSG_01196
Aque_229282
Mvib_comp3686_c0_seq1_fr5
Awhi_Contig17957
Pgem_Contig49795
Awhi_Contig23971
Awhi_Contig57820
Pgem_Contig51197
Apar_comp23853
Apar_comp24261
Cfra_g3780
Sarc_SARC_12941
Sarc_SARC_13968
Sarc_SARC_15953
Clim_Contig1231
Rory_15113
Rory_6035
Pbla_Phybl2_136641
Pbla_Phybl2_137096
Rory_16422
Rory_10110
Rory_6086
Rory_15185
Pbla_Phybl2_154650
Pbla_Phybl2_185687
Rory_13880
Mver_MVEG_00810
Mver_MVEG_02983
Tmel_XP_002838719
AoQ2UTJ
Tmel_XP_002838720
Umay_XP_760702
Cneo_XP_570120
Ccin_CC1G_03780
Spun_SPPG_01285
Bden_Batde5_13697
Amac_AMAG_09783
Amac_AMAG_04247
Spom_NP_588492_1
Spom_NP_596233_1
Awhi_Contig78316
Awhi_Contig63236
Pgem_Contig73864
Pgem_Contig80832
Awhi_Contig10582
Pgem_Contig70571
Pgem_Contig6201
Pgem_Contig63754
Pgem_Contig44864
Pgem_Contig68573
Apar_comp23489
Apar_comp24301
Cowc_CAOG_05212
Clim_Contig5299
Awhi_Contig71392
Awhi_Contig68299
Pgem_Contig17235
Pgem_Contig9535
Pgem_Contig38551
Pgem_Contig77481
Sarc_SARC_07634
Cfra_g1016
Apar_comp20627
Dpul_221760
Hsap_NP_001157207
Nvec_1247808
Ctel_222718
Acoe_063_00017
Acoe_063_00023
Atha_AT4G27370
Atha_AT5G54280
Sbic_02g036390
Acoe_008_00012
Atha_AT1G50360
Atha_AT3G19960
Atha_AT1G42680
Acoe_035_00049
Sbic_01g018770
Php_XP_001755337
Php_XP_001777862
Php_XP_001774306
Php_XP_001775964
Php_XP_001768683
Smol_XP_002975651
Smol_XP_002978834
Smol_XP_002972734
Smol_XP_002993349
Crei_XP_001696752
Vcar_XP_002956099
Cvar_EFN52211

Figure S4

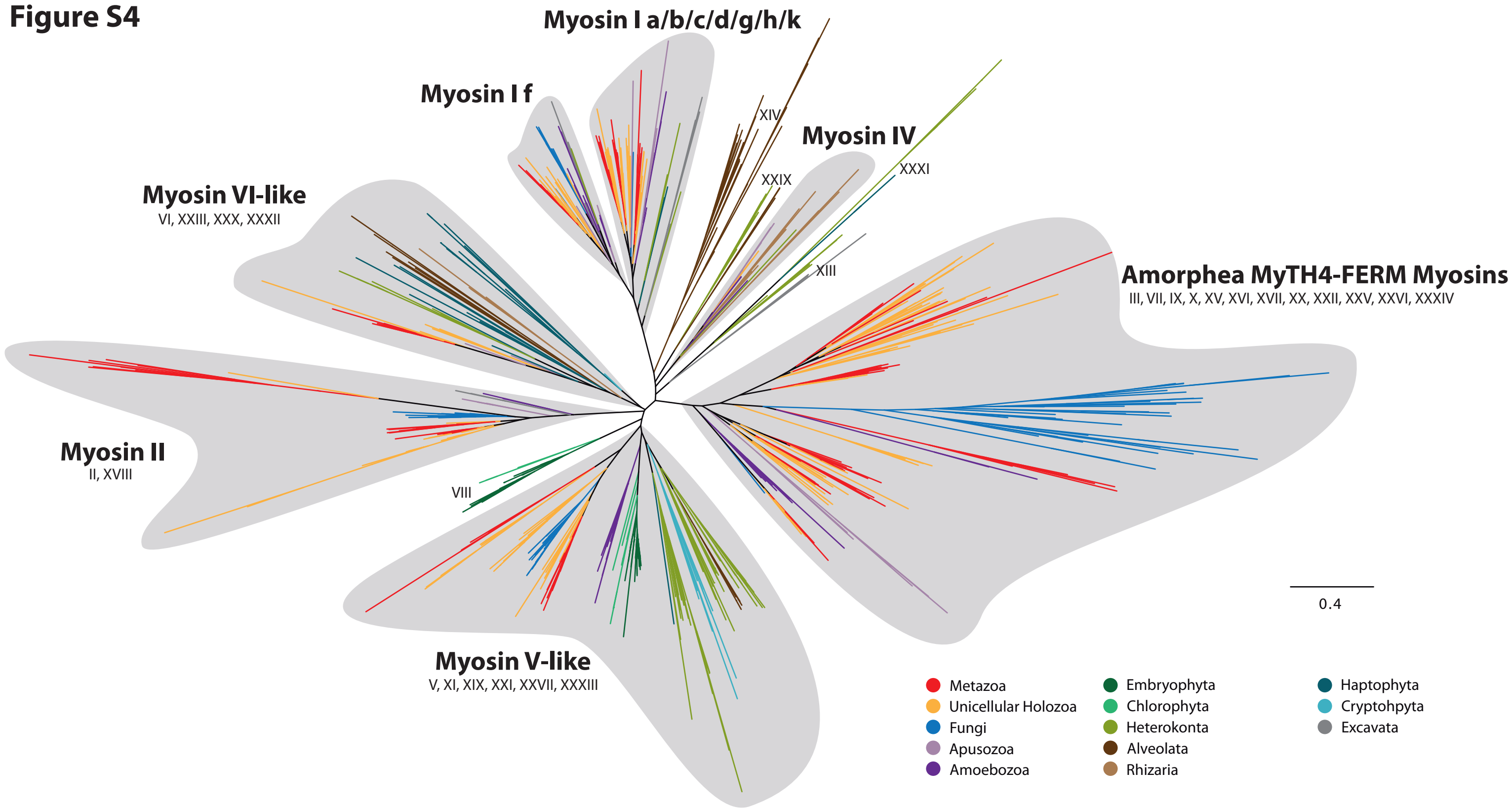

Myosin I a/b/c/d/g/h/k
Myosin I f
Myosin IV
XIV
XXIX
XXXI
XIII
Myosin VI-like
VI, XXIII, XXX, XXXII
Amorphea MyTH4-FERM Myosins
III, VII, IX, X, XV, XVI, XVII, XX, XXII, XXV, XXVI, XXXIV
Myosin II
II, XVIII
VIII
Myosin V-like
V, XI, XIX, XXI, XXVII, XXXIII
0.4
Metazoa
Unicellular Holozoa
Fungi
Apusozoa
Amoebozoa
Embryophyta
Chlorophyta
Heterokonta
Alveolata
Rhizaria
Haptophyta
Cryptohpyta
Excavata

Figure S5

A

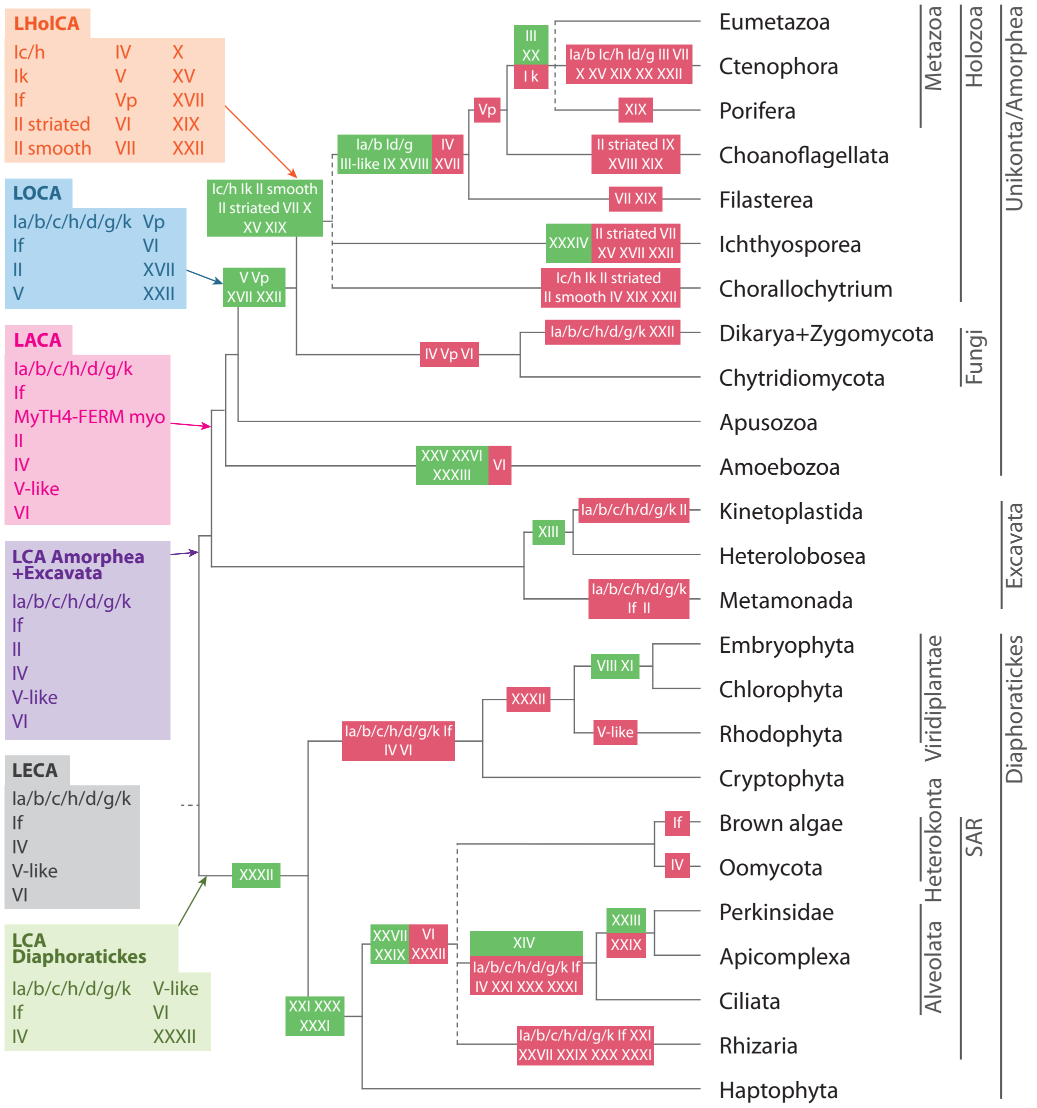
LHolCA
Ic/h IV X
Ik V XV
If Vp XVII
II striated VI XIX
II smooth VII XXII
LOCA
Ia/b/c/h/d/g/k Vp
If VI
II XVII
V XXII
LACA
Ia/b/c/h/d/g/k
If
MyTH4-FERM myo
II
IV
V-like
VI
LCA Amorphea +Excavata
Ia/b/c/h/d/g/k
If
II
IV
V-like
VI
LECA
Ia/b/c/h/d/g/k
If
IV
V-like
VI
LCA Diaphoratickes
Ia/b/c/h/d/g/k V-like
If VI
IV XXXII
Ic/h Ik II smooth II striated VII X XV XIX
V Vp XVII XXII
Ia/b Id/g III-like IX XVIII
IV XVII
Vp
III XX
I k
Ia/b Ic/h Id/g III VII X XV XIX XX XXII
XIX
II striated IX XVIII XIX
VII XIX
XXXIV
II striated VII XV XVII XXII
Ic/h Ik II striated II smooth IV XIX XXII
IV Vp VI
Ia/b/c/h/d/g/k XXII
XXV XXVI XXXIII
VI
XIII
Ia/b/c/h/d/g/k II
Ia/b/c/h/d/g/k If II
VIII XI
XXXII
V-like
Ia/b/c/h/d/g/k If IV VI
XXXII
If
IV
XXIII
XXIX
XIV
Ia/b/c/h/d/g/k If IV XXI XXX XXXI
XXVII XXIX
VI XXXII
XXI XXX XXXI
Ia/b/c/h/d/g/k If XXI XXVII XXIX XXX XXXI
Eumetazoa
Ctenophora
Porifera
Choanoflagellata
Filasterea
Ichthyosporea
Chorallochytrium
Dikarya+Zygomycota
Chytridiomycota
Apusozoa
Amoebozoa
Kinetoplastida
Heterolobosea
Metamonada
Embryophyta
Chlorophyta
Rhodophyta
Cryptophyta
Brown algae
Oomycota
Perkinsidae
Apicomplexa
Ciliata
Rhizaria
Haptophyta
Metazoa
Holozoa
Unikonta/Amorphea
Fungi
Excavata
Viridiplantae
Diaphoratickes
Heterokonta
SAR
Alveolata

B

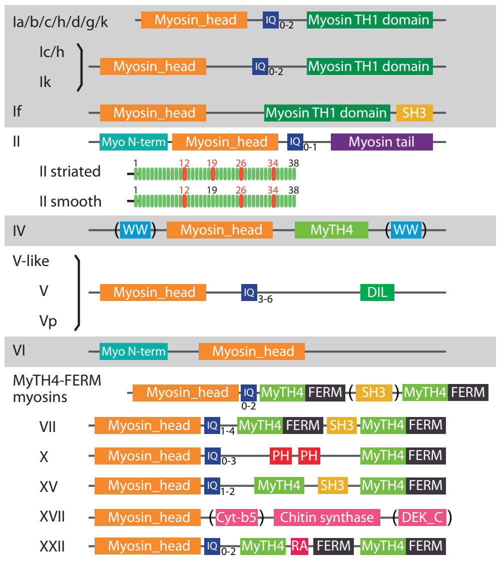
Ia/b/c/h/d/g/k
Myosin_head
IQ
0-2
Myosin TH1 domain
Ic/h
Ik
If
SH3
II
Myo N-term
0-1
Myosin tail
II striated
II smooth
1 12 19 26 34 38
IV
WW
MyTH4
V-like
V
Vp
3-6
DIL
VI
MyTH4-FERM myosins
FERM
VII
1-4
X
0-3
PH
XV
1-2
XVII
Cyt-b5
Chitin synthase
DEK_C
XXII
RA

Figure S6

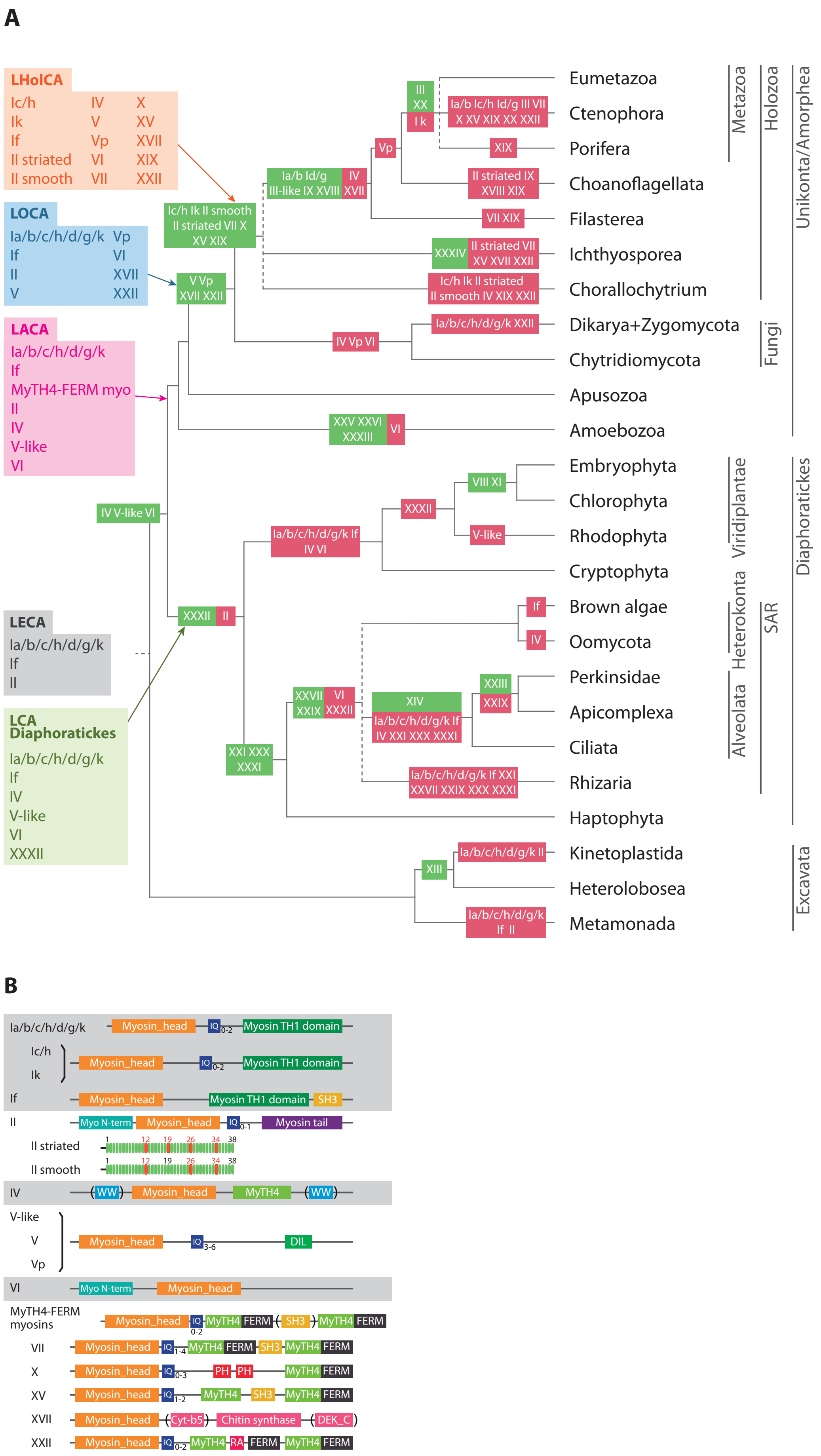
A
LHolCA
Ic/h IV X
Ik V XV
If Vp XVII
II striated VI XIX
II smooth VII XXII
LOCA
Ia/b/c/h/d/g/k Vp
If VI
II XVII
V XXII
LACA
Ia/b/c/h/d/g/k
If
MyTH4-FERM myo
II
IV
V-like
VI
LECA
Ia/b/c/h/d/g/k
If
II
LCA Diaphoratickes
Ia/b/c/h/d/g/k
If
IV
V-like
VI
XXXII
III XX
I k
Ia/b Ic/h Id/g III VII X XV XIX XX XXII
XIX
Vp
Ia/b Id/g III-like IX XVIII
IV XVII
II striated IX XVIII XIX
VII XIX
Ic/h Ik II smooth II striated VII X XV XIX
XXXIV
II striated VII XV XVII XXII
Ic/h Ik II striated II smooth IV XIX XXII
V Vp XVII XXII
Ia/b/c/h/d/g/k XXII
IV Vp VI
XXV XXVI XXXIII
VI
VIII XI
XXXII
V-like
Ia/b/c/h/d/g/k If IV VI
IV V-like VI
XXXII
II
If
IV
XXIII
XXIX
XXVII XXIX
VI XXXII
XIV
Ia/b/c/h/d/g/k If IV XXI XXX XXXI
XXI XXX XXXI
Ia/b/c/h/d/g/k If XXI XXVII XXIX XXX XXXI
XIII
Ia/b/c/h/d/g/k II
Ia/b/c/h/d/g/k If II
Eumetazoa
Ctenophora
Porifera
Choanoflagellata
Filasterea
Ichthyosporea
Chorallochytrium
Dikarya+Zygomycota
Chytridiomycota
Apusozoa
Amoebozoa
Embryophyta
Chlorophyta
Rhodophyta
Cryptophyta
Brown algae
Oomycota
Perkinsidae
Apicomplexa
Ciliata
Rhizaria
Haptophyta
Kinetoplastida
Heterolobosea
Metamonada
Metazoa
Holozoa
Unikonta/Amorphea
Fungi
Viridiplantae
Heterokonta
Alveolata
SAR
Diaphoratickes
Excavata
B
Ia/b/c/h/d/g/k
Myosin_head
IQ 0-2
Myosin TH1 domain
Ic/h
Ik
If
SH3
II
Myo N-term
IQ 0-1
Myosin tail
II striated
II smooth
1 12 19 26 34 38
IV
WW
MyTH4
V-like
V
Vp
IQ 3-6
DIL
VI
MyTH4-FERM myosins
FERM
VII
IQ 1-4
X
IQ 0-3
PH
XV
IQ 1-2
XVII
Cyt-b5
Chitin synthase
DEK_C
XXII
RA

Table S1. Nomenclatural equivalences between this study and two previous studies (Odronitz *et al.* 2007 and Foth *et al.* 2006). It is important to note that the fact that we respect a name does not mean that the family includes the very same lineages, but usually an extended repertoire of species (Figure 1). Some classes merge previous independent groups, while others myosin VII or myosin V have been split.

This study		Odronitz 2007	Foth 2006	Notes
**1**	I	I	I	
**2**	II	II	II	
**3**	III	III	III	
**4**	IV	IV	IV	
**5**	V (w/o Amoebozoa)	V	V	We rename amoebozoan homologs of V as XXXIII myosins (see below). We also find a new group of V myosins lost in animals and fungi (Vb).
**6**	VI (w/o Alveolata)	VI	VI	Foth 2006 found apicomplexan MyoK and MyoJ branching together with myosin VI. In our analysis, these sequences appear to be related to myosin XXIII (alveolate-specific) an, together, only very weakly related to myosin VI. For this reason, we include all of them inside the XXIII class and suggest its putative relation with myosin VI in Figure S1.
**7**	VII	VII	VII (w/o Amoebozoa)	We rename amoebozoan VII myosins as XXV (see below).
**8**	VIII	VIII	VIII	
**9**	IX	IX	IX	
**10**	X	X	X	
**11**	XI	XI	XI	
**13**	XIII	XIII	XXI	
**14**	XIV	XIV + XXV	XIV	
**15**	XV	XV	XV	
**16**	XVI	XVI	XVI	
**17**	XVII	XVII	XVII	
**18**	XVIII	XVIII	XVIII	
**19**	XIX	XIX	XIX	
**20**	XX	XX	XX	
**21**	XXI	Not present	Not present	We re-use the name XXI, in Odronitz 2007 it included Toxoplasma sequences that we identify as a clear myosin III homolog (Figure S1).
**22**	XXII	XXII	Not present	
**23**	XXIII	XXIII+XXVI	XXIII	
**25**	XXV	Not present	Amoebozoa VII	We re-use the class name XXV, in Odronitz 2007 it included a Drosophila sequence that we identify as a clear myosin XIV homolog (see Figure S1).
**26**	XXVI	?	?	
**27**	XXVII	XXVII + XXIV	XXII	
**29**	XXIX	XXIX	Not present	
**30**	XXX	XXX	Not present	
**31**	XXXI	XXXI+XXXIII	Not present	
**32**	XXXII	Not present	Not present	We re-use the class name XXXIII, as in Odronitz 2007 includes an extremely derived Phytophtora sequence that doesn't have a clear myosin domain.
**33**	XXXIII	Amoebozoa V	Amoebozoa V	We re-use the name XXXIII, as the sequences under the class 33 in Odronitz 2007 are clearly part of myosin class XXXI.
**34**	XXXIV	Not present	Not present	
**Techamonas orphan**	No name	Not present	Not present	
**Bigellowiella orphan**	No name	Not present	Not present	
**Guillardia orphan**	No name	Not present	Not present	
**Emiliania orphan**	No name	Not present	Not present	

Table S2. Taxon sampling used in this study, including the taxonomic classification, the acronym used in the present study (between brackets) and the source of the proteome data.

Taxonomic classification		Species	Source
**METAZOA**		*Homo sapiens (Hsap)*	Ensembl
		Daphnia pulex (Dpul)	
		Drosophila melanogaster (Dmel)	Flybase *http://flybase.org/*
		Capitella teleta (Ctel)	JGI
		Nematostella vectensis (Nvec)	NCBI
		Mnemiopsis leidyi (Mlei)	*M. leidyi* Genome Project webpage *http://research.nhgri.nih.gov/mnemiopsis/*
		Oscarella carmela (Ocar)	Courtesy of Scott Nichols
		Amphimedon queenslandica (Aque)	NCBI
**CHOANOFLAGELLATA**		*Monosiga brevicollis (Mbre)*	NCBI
		Salpingoeca rosetta (Sros)	Broad Institute *http://www.broadinstitute.org/annotation/genome/multicellularity_project/MultiHome.html*
**FILASTEREA**		*Capsaspora owczarzaki (Cowc)*	Broad Institute
		Ministeria vibrans (Mvib)	RNASeq data
**ICHTHYOSPOREA**		*Sphaeroforma arctica (Sarc)*	Broad Institute
		Creolimax fragrantissima (Cfra)	*Ab initio protein prediction*
		Abeoforma whisleri (Awhi)	RNASeq data
		Pirum gemmata (Pgem)	RNASeq data
		Amoebidium parasiticum (Apar)	RNASeq data
	Incertae sedis	*Corallochytrium limacisporum (Clim)*	RNASeq data
**FUNGI**	Ascomycota	*Aspergillus oryzae (Aory)*	NCBI
		Schizosaccharomyces pombe (Spom)	NCBI
		Tuber melanosporum (Tmel)	NCBI
	Basidiomycota	*Cryptococcus neoformans (Cneo)*	NCBI
		Coprinopsis cinerea (Ccin)	Broad Institute
		Ustilago maydis (Umay)	NCBI
	Incertae sedis	*Mortierella verticillata (Mver)*	Broad Institute
	Mucoromycotina	*Phyomyces blakesleeanus (Pbla)*	JGI
		Rhizopus oryzae (Rory)	Broad Institute
	Blastocladio-mycota	*Allomyces macrogynus (Amac)*	Broad Institute
	Chytridiomycota	*Batrachochytrium dendrobatidis (Bden)*	JGI
		Spizellomyces punctatus (Spun)	Broad Institute
**APUSOZOA**		*Thecamonas trahens (Ttra)*	Broad Institute
**AMOEBOZOA**	Mycetozoa	*Dictyostelium discoideum (Ddis)*	NCBI
		Polysphondylium pallidum (Ppal)	NCBI
	Archamoeba	*Entamoeba histolytica (Ehis)*	Wellcome Trust Sanger Institute *http://www.sanger.ac.uk/resources/downloads/protozoa/entamoeba.html*
	Centramoebida	*Acanthamoeba castellanii (Acas)*	*Ab initio protein prediction*
**VIRIDIPLANTAE**	Embryophyta	*Arabidopsis thaliana (Atha)*	NCBI
		Aquilegia coerulea (Acoe)	Phytozome (JGI)
		Sorghum bicolor (Sbic)	Phytozome (JGI)
		Selaginella moellendorffii (Smoe)	NCBI
		Physcomitrella patens (Ppat)	NCBI
	Chlorophyta	*Chlamydomonas reinhardtii (Crei)*	NCBI
		Volvox cartieri (Vcar)	NCBI

		Chlorella variabilis (Cvar)	NCBI
		Ostreococcus tauri (Otau)	NCBI
	Rhodophyta	*Cyanidioschyzon merolae (Cmer)*	*C. merolae* Genome Project webpage *http://merolae.biol.s.u-tokyo.ac.jp/*
		Chondrus crispus (Ccris)	Courtesy of Jonas Collén
**HETERO-KONTA**	Brown algae/ Phaeophyceae	*Ectocarpus siliculosus (Esil)*	EMBL
		Nannochloropsis gaditana (Ngad)	*N. gaditana* Genome Project webpage *http://nannochloropsis.genomeprojectsolutions-databases.com/*
		Phaeodactylum tricornutum (Ptri)	NCBI
		Thalassiossira pseudonana (Tpse)	NCBI
	Oomycota	*Phytophthora infestans (Pinf)*	NCBI
**ALVEOLATA**	Apicomplexa	*Toxoplasma gondii (Tgon)*	NCBI
		Plasmodium falciparum (Pfal)	Uniprot
	Ciliophora	*Paramecium tetraurelia (Ptet)*	Uniprot
		Tetrahymena thermophila (Tthe)	NCBI
	Perkinsidae	*Perkinsus marinus (Pmar)*	NCBI
**RHIZARIA**		*Bigelowiella natans (Bnat)*	JGI
**HAPTOPHYTA**		*Emiliania huxleyi (Ehux)*	JGI
**CRYPTOPHYTA**		*Guillardia theta (Gthe)*	JGI
**EXCAVATA**	Heterolobosea	*Naegleria gruberi (Ngru)*	NCBI
	Kinetoplastida	*Trypanosoma cruzi (Tcru)*	NCBI
		Leishmania major (Lmaj)	NCBI
	Metamonada	*Trichomonas vaginalis (Tvag)*	NCBI
		Giardia lamblia (Glam)	NCBI
